# Real-world toxicity and discontinuation risk in ibrutinib therapy: a retrospective cohort study

**DOI:** 10.3389/fmed.2026.1897420

**Published:** 2026-09-14

**Authors:** Wid M. Alyamani, Attiah Khobrani, Fawaz O. Alenazy, Yahya A. Alzahrani, Maan H. Harbi

**Affiliations:** 1 Pharmaceutical Care, King Abdullah Medical City, Makkah, Saudi Arabia; 2 Department of Clinical Laboratory Sciences, College of Applied Medical Sciences, Jouf University, Sakaka, Saudi Arabia; 3 Department of Pharmacology, Faculty of Medicine, King Abdulaziz University, Rabigh, Saudi Arabia; 4 Pharmacology and Toxicology Department, College of Pharmacy, Umm Al-Qura University, Makkah, Saudi Arabia

**Keywords:** adverse events, Bruton tyrosine kinase inhibitor, chronic lymphocytic leukemia, ibrutinib, ischemic heart disease, real-world evidence, treatment discontinuation, drug safety

## Abstract

**Background:**

Ibrutinib is widely used in B-cell malignancies, yet real-world safety data remain heterogeneous, particularly in underrepresented regional populations.

**Objective:**

To characterize the adverse-event profile and severity and explore factors associated with Grade 3–5 toxicity and permanent treatment discontinuation among patients receiving ibrutinib in routine clinical practice.

**Methods:**

A retrospective cohort study was conducted at King Abdullah Medical City, Makkah, Saudi Arabia, with follow-up through December 2025. Adult patients with B-cell malignancies who received ibrutinib and had sufficient clinical documentation for safety-outcome ascertainment were included. The primary endpoint was the occurrence of at least one Grade 3–5 adverse event according to CTCAE v5.0. Secondary outcomes included dose modifications, permanent treatment discontinuation, factors associated with toxicity, and temporal adverse-event patterns.

**Results:**

Among 57 patients, all 57 (100%) experienced at least one adverse event and 41 (71.9%) experienced at least one Grade 3–5 adverse event. Neutropenia and hypertension were the most frequent severe toxicities. Dose modification occurred in 47 patients (82.5%), and permanent discontinuation occurred in 17 (29.8%). In reduced Firth penalized-likelihood models adjusted for starting-dose category, pre-existing ischemic heart disease was associated with permanent discontinuation (aOR 6.25, 95% profile-likelihood CI 1.29–39.19; *p* = 0.023), while creatinine clearance <60 mL/min was associated with Grade 3–5 toxicity (aOR 5.53, 95% profile-likelihood CI 1.26–38.46; *p* = 0.022). A patient-level omnibus permutation test indicated a difference in adverse-event cluster distributions between the early (≤90 days) and late (>90 days) periods (*p* = 0.004), although an equal-window sensitivity analysis comparing days 0–90 with days 91–180 was not significant (*p* = 0.240).

**Conclusion:**

Ibrutinib was associated with substantial toxicity and frequent treatment modification in routine clinical practice. Pre-existing ischemic heart disease and impaired renal function emerged as exploratory markers of treatment vulnerability. The temporal findings support continued surveillance throughout therapy but do not establish a phase-specific monitoring strategy.

## 1 Introduction

Ibrutinib, a first-in-class covalent Bruton tyrosine kinase inhibitor (BTKi), has transformed the treatment landscape for B-cell malignancies, including chronic lymphocytic leukemia (CLL), small lymphocytic lymphoma (SLL), and Waldenström's macroglobulinemia (WM). Its clinical efficacy has been established in pivotal trials of both relapsed/refractory and treatment-naïve CLL/SLL; however, because ibrutinib is generally administered continuously until disease progression or unacceptable toxicity, long-term tolerability is a central determinant of treatment success (1, 2).

The safety profile of ibrutinib is clinically distinctive. Bleeding, atrial fibrillation, hypertension, infections, and cytopenias have been consistently reported across randomized trials, long-term follow-up analyses, and real-world cohorts (3–6). These adverse events (AEs) are not merely descriptive safety outcomes; they may lead to treatment interruption, dose reduction, permanent discontinuation, hospitalization, or switching to alternative BTK inhibitors. More selective second-generation BTK inhibitors have expanded the available treatment options for CLL/SLL and may offer improved tolerability for selected patients. In the randomized ELEVATE-RR trial, acalabrutinib demonstrated noninferior progression-free survival compared with ibrutinib, with lower incidences of atrial fibrillation and hypertension (7). In this evolving therapeutic landscape, characterizing the real-world toxicity of ibrutinib remains important for treatment selection, risk stratification, longitudinal monitoring, and decisions regarding switching therapy. Accordingly, evaluating severe toxicity and discontinuation risk is essential when translating trial efficacy into routine clinical practice.

Real-world safety data are particularly important because patients treated outside clinical trials often differ from trial populations in age, comorbidity burden, baseline organ function, concomitant medication exposure, and intensity of monitoring (6, 8, 9). Cardiovascular comorbidity is especially relevant for ibrutinib-treated patients, given the recognized association of ibrutinib with atrial fibrillation, hypertension, bleeding, and other cardiovascular complications (4, 5). Similarly, impaired renal function may identify a clinically vulnerable subgroup with higher susceptibility to hematologic, infectious, or treatment-related complications, even though ibrutinib itself is predominantly hepatically metabolized.

In addition to identifying who is at risk, an important unanswered question is when specific toxicities are most likely to occur. Prior long-term analyses have described changes in the frequency of selected AEs over time, but fewer studies have evaluated whether organ-system AE clusters follow distinct early vs. late temporal patterns that can be operationalized into monitoring strategies (3, 4). A temporal framework may be particularly useful in routine practice, where surveillance must be practical, risk-adapted, and sustained over prolonged therapy.

Regional real-world evidence from the Middle East remains limited, despite potential differences in patient characteristics, comorbidity patterns, referral pathways, and treatment-monitoring practices. Local real-world data are therefore needed to contextualize international safety findings and to identify clinically relevant risk factors in Saudi and Middle Eastern practice settings. This study aimed to characterize the real-world AE profile of ibrutinib-treated patients with B-cell malignancies at a tertiary center in Saudi Arabia, with emphasis on Grade ≥3 toxicity, dose modification, and permanent discontinuation. The study further sought to identify baseline factors associated with severe toxicity and discontinuation, evaluate patient-level temporal patterns of adverse-event clusters, and explore cumulative AE burden and dose-modification patterns.

## 2 Materials and methods

### 2.1 Study design

This was a single-center retrospective cohort study based on chart review and electronic health-record data from King Abdullah Medical City, Makkah, Saudi Arabia. Eligible patients initiated ibrutinib between March 2016 and February 2025, and clinical follow-up was assessed through December 1, 2025.

### 2.2 Study population

Adults aged ≥18 years with a documented B-cell malignancy who received ibrutinib and had sufficient post-baseline documentation for adverse-event and treatment-outcome ascertainment were eligible. Patients with CLL, CLL/SLL, Waldenström's macroglobulinemia, mantle cell lymphoma, and other documented B-cell malignancies were included.

Patients with fewer than 30 days of exposure were not excluded solely because of early treatment cessation or switching. Such patients remained eligible when available documentation permitted ascertainment of ibrutinib exposure, adverse events, and management actions. Records were excluded when ibrutinib was initiated outside the study center without subsequent follow-up, exposure or outcome documentation was inadequate, another covalent BTK inhibitor was used concurrently at ibrutinib initiation, or early switching precluded reliable attribution of adverse events and treatment outcomes.

### 2.3 Endpoints

#### 2.3.1 Primary endpoint

The primary endpoint was the proportion of patients experiencing at least one Grade ≥3 AE occurring from ibrutinib initiation until treatment discontinuation, death, or end of follow-up, according to the Common Terminology Criteria for AEs, version 5.0 (CTCAE v5.0) during ibrutinib therapy.

#### 2.3.2 Secondary endpoints

- Rate of ibrutinib dose modifications attributable to adverse events, including temporary interruption, dose reduction, and permanent discontinuation.
- Identification of baseline patient and disease characteristics associated with (a) Grade ≥3 adverse events and (b) ibrutinib discontinuation using univariable and multivariable analyses.
- Evaluation of temporal adverse-event patterns by comparing organ-system AE cluster distributions between early (≤90 days) and late (>90 days) treatment periods.
- Time-to-event analyses including time to first Grade ≥3 adverse event and time to treatment discontinuation stratified by significant baseline predictors.

#### 2.3.3 Exploratory endpoints

- Association between cumulative adverse-event burden and Grade ≥3 toxicity.
- Dose-reduction patterns and recurrence of subsequent adverse events.
- Relationship between ibrutinib hold duration, adverse-event characteristics, and permanent discontinuation.

### 2.4 Data collection

A total of 72 potentially eligible records were screened. Fifteen were excluded: six because of short or inadequately documented ibrutinib exposure, including early treatment switching; eight because treatment administration, follow-up, or clinical information was insufficient for reliable safety-outcome ascertainment; and one because the documented events occurred after the patient was no longer receiving ibrutinib. The final analytic cohort comprised 57 patients. Baseline demographic and clinical information was not consistently available for the excluded records; therefore, formal comparison between included and excluded patients was not possible.

Patient data were extracted retrospectively from the EHR by the principal investigator and co-investigators. Data were entered into standardized electronic forms with each patient assigned a unique study code. Identifying information was stored separately in a secure, password-protected log accessible only to the principal investigator. Adverse events were ascertained through structured review of hematology and oncology physician progress notes, nursing documentation, emergency department and inpatient admission and discharge records, laboratory results, and pharmacy documentation of dose interruption, dose reduction, or discontinuation. For each potential AE, the event description, onset date, relevant objective findings, CTCAE grade, management action, and outcome were abstracted. Laboratory-defined toxicities were graded directly from available laboratory values using CTCAE v5.0 thresholds, whereas clinically defined toxicities were graded using the documented symptoms, functional impact, interventions required, and need for hospitalization. When the same clinical episode was documented in multiple sources, the records were reconciled and the episode was counted once using the earliest documented onset date and the highest grade supported by the available documentation. Events without sufficient information to establish their occurrence or severity were not included. The following data were collected:

- Demographics: age at treatment initiation, sex, body mass index (BMI), and smoking status.
- Disease characteristics: diagnosis; cytogenetic abnormalities [del(13q), del(11q), trisomy 12, del(17p), TP53, IGHV mutation status].
- Baseline comorbidities: hypertension (HTN), diabetes mellitus (DM), ischemic heart disease (IHD), chronic kidney disease (CKD), and epilepsy.
- Baseline laboratory values: serum creatinine (mg/dL).
- Treatment details: ibrutinib starting dose; concomitant medications (anticoagulants, antiplatelets, CYP3A4 inhibitors/inducers, immunosuppressants, vaccines).
- AEs: type, date of onset, CTCAE grade, cumulative ibrutinib exposure at onset, action taken on ibrutinib, hold duration, dose changes, and outcome.

### 2.5 Ethical considerations

The study was approved by the Institutional Review Board of King Abdullah Medical City (IRB approval number 25-1490). A consent waiver was granted given the retrospective observational design involving secondary data from medical records with no more than minimal risk. All data were de-identified prior to analysis. The study was conducted in accordance with the Declaration of Helsinki and institutional policies on data confidentiality.

### 2.6 Variable definitions

#### 2.6.1 Adverse event grading

All adverse events were graded according to the National Cancer Institute Common Terminology Criteria for Adverse Events (CTCAE) version 5.0 (10).

#### 2.6.2 Creatinine clearance

Creatinine clearance (CrCl) was estimated using the Cockcroft–Gault equation (11):

CrCl(mL/min)=[(140−ageinyears)×bodyweight(kg)]/[72×serumcreatinine(mgdL)]

For female patients, the result was multiplied by 0.85 to account for lower creatinine production due to reduced muscle mass. Renal function was categorized according to the FDA Guidance for Industry and KDIGO recommendations (12, 13):

- Normal: CrCl ≥90 mL/min.
- Mild impairment: CrCl 60–89 mL/min.
- Moderate impairment: CrCl 30–59 mL/min.
- Severe impairment: CrCl <30 mL/min.

For analytical purposes, renal impairment was defined as Cockcroft–Gault creatinine clearance <60 mL/min. This combined the moderate and severe categories because only one patient had severe renal impairment.

#### 2.6.3 Body mass index classification

Body mass index (BMI) was calculated as weight (kg) divided by height squared (m^2^) and categorized according to the World Health Organization (WHO) classification (14):

- Underweight: BMI <18.5 kg/m^2^.
- Normal weight: BMI 18.5–24.9 kg/m^2^.
- Overweight: BMI 25.0–29.9 kg/m^2^.
- Obese: BMI ≥30.0 kg/m^2^.

For analytical purposes, underweight and normal-weight categories were merged due to a single underweight patient (*n* = 1).

#### 2.6.4 Age classification

The primary age dichotomy of <65 vs. ≥65 years was selected to align with the age stratification used in major ibrutinib clinical trials, including RESONATE and RESONATE-2, in which 65 years is the accepted threshold for elderly patients in CLL/SLL. A three-tier subgroup classification (<50, 50–64, ≥65 years) was used for exploratory analyses (1, 2).

#### 2.6.5 Adverse event cluster classification

Adverse events were grouped into 11 prespecified organ-system clusters using the Medical Dictionary for Regulatory Activities (MedDRA) System Organ Class hierarchy, with study-specific adaptation to reflect clinically relevant ibrutinib-associated toxicity domains: hematologic, cardiac, infectious, bleeding, gastrointestinal, dermatologic, hepatic, general, metabolic, renal, and neurological (15). This framework was informed by adverse-event categories of special interest reported in the RESONATE-2 long-term follow-up and the FDA prescribing information for ibrutinib (3). Individual adverse events were assigned to the most clinically appropriate predefined cluster according to MedDRA terminology.

#### 2.6.6 Early vs. late AE classification

A prespecified 90-day landmark was used to classify AEs into early (≤90 days of cumulative ibrutinib exposure) and late (>90 days) periods. This temporal cutoff was selected based on published ibrutinib safety data showing that many hematologic and bleeding toxicities manifest within the first 3 months, while cardiac and infectious complications tend to emerge later (3, 4). The 90-day threshold is also consistent with the time frame used in the integrated safety analysis of ibrutinib pivotal studies for evaluating early treatment tolerability (4).

#### 2.6.7 Dose modification definitions

Dose interruption was defined as any temporary cessation of ibrutinib (including events where ibrutinib was subsequently resumed at a lower dose). Dose reduction was defined as any decrease in ibrutinib dose (with or without prior interruption). Dose-reduction events were counted only when the documented post-event dose was lower than the pre-event dose. Increases or re-escalations were not classified as dose reductions. Permanent discontinuation was defined as complete and permanent cessation of ibrutinib due to AEs. Categories are not mutually exclusive at the patient level, as a single patient may have experienced multiple types of dose modifications during treatment.

#### 2.6.8 AE burden definition

AE burden was defined as the total number of AEs experienced per patient during the entire ibrutinib treatment course. For descriptive purposes, AE burden was categorized as low (1–2 AEs), moderate (3–4 AEs), or high (≥5 AEs). This categorization was exploratory and developed *post hoc* based on the observed distribution of AEs in the cohort. The continuous AE count was used for inferential analysis.

### 2.7 Statistical analysis

Data management and initial descriptive analyses were performed using IBM SPSS Statistics version 25 (IBM Corp., Armonk, NY, USA). Kaplan–Meier results were initially examined using GraphPad Prism version 10 (GraphPad Software, San Diego, CA, USA). All final multivariable, survival, temporal, sensitivity, and figure-specific analyses were generated or independently reproduced using R version 4.6.1. Final statistical figures were generated in R using ggplot2 version 4.0.3. A two-sided *p*-value <0.05 was considered statistically significant.

Continuous variables were assessed for normality using the Shapiro–Wilk test. Normally distributed variables were summarized as mean and standard deviation, whereas non-normally distributed variables were presented as median and interquartile range. Categorical variables were summarized as frequencies and percentages.

Categorical variables were compared using the chi-square test or Fisher's exact test, as appropriate. Odds ratios and 95% confidence intervals were reported for binary associations. The Mann–Whitney *U* test was used to compare non-normally distributed continuous variables between two independent groups, and the Kruskal–Wallis test was used for comparisons across three or more groups.

Because of the modest cohort size and limited number of outcome events, adjusted analyses were intentionally restricted to two parsimonious models. Firth penalized-likelihood logistic regression was used to reduce small-sample and sparse-data bias. The permanent-discontinuation model included pre-existing ischemic heart disease as the principal exposure and starting-dose category as a clinically relevant potential confounder. The Grade ≥3 adverse-event model included impaired renal function, defined as Cockcroft–Gault creatinine clearance <60 mL/min, and starting-dose category. The 420-mg starting dose was the reference category. Adjusted odds ratios, profile-likelihood 95% confidence intervals, and penalized-likelihood *p*-values were reported. Apparent model discrimination was assessed using the C-index, equivalent to the area under the receiver operating characteristic curve, with 95% confidence intervals. Firth models were fitted using the logistf package version 1.26.1, and C-indices were calculated using pROC version 1.19.0.1. Because model performance was evaluated in the derivation cohort without external validation, the models were interpreted as exploratory association models rather than clinical prediction models.

Time to permanent discontinuation was evaluated using Kaplan–Meier estimates and the log-rank test. Hazard ratios and 95% confidence intervals were estimated using univariable Cox proportional-hazards models fitted with the survival package version 3.8-6. Patients without permanent discontinuation were censored at their last documented follow-up.

Temporal adverse-event patterns were evaluated at the patient level. For each patient and organ-system cluster, binary indicators denoted the occurrence of at least one adverse event during the early period (days 0–90) and late period (>90 days). The global difference in paired cluster profiles was evaluated using a within-patient permutation test in which complete early and late profiles were randomly exchanged within patients across 100,000 permutations. Cluster-specific early-vs.-late comparisons used two-sided exact McNemar tests, with *p*-values adjusted across the 11 clusters using the Holm method. To address the unequal opportunity for event accumulation in the primary comparison, an equal-window sensitivity analysis compared days 0–90 with days 91–180 among patients who remained under observation without discontinuation or death through day 180.

Starting-dose groups were compared with respect to age, ischemic heart disease, renal impairment, Grade ≥3 toxicity, and permanent discontinuation. Age was compared using the Kruskal–Wallis test, and categorical variables were compared using Fisher's exact test. Starting-dose category was retained in both Firth models because of its pharmacological relevance and potential association with clinician-directed treatment selection.

Calendar-era analyses compared patients initiating treatment before 2022 with those initiating during 2022–2025. Grade ≥3 toxicity, permanent discontinuation, and initiation below 420 mg were compared using Fisher's exact test. Because follow-up opportunities differed between eras, time to permanent discontinuation and time to first Grade ≥3 adverse event were additionally examined using univariable Cox models and log-rank tests.

Cumulative adverse-event counts were compared using the Mann–Whitney *U* test. For patients with multiple documented treatment holds, identical hold and resumption dates were treated as a single hold episode. For patient-level comparisons, the median hold duration was calculated for each patient to avoid treating repeated holds within the same patient as independent observations.

Sensitivity analyses included restriction to patients with CLL/CLL-SLL and inclusion of the excluded early-switch patient as an additional patient with no documented adverse event. Analyses used complete available cases; no missing values were imputed. Analyses involving variables with missing data were restricted to patients with available values. No missing values were imputed.

## 3 Results

### 3.1 Baseline characteristics

A total of 57 patients met the inclusion criteria and were included in the analysis. The median age at ibrutinib initiation was 64 years (IQR 56–70), and 28 patients (49.1%) were aged ≥65 years. Most patients were male (39, 68.4%). Nearly all patients had CLL or CLL/SLL (94.7%). Baseline comorbidity burden was substantial, with diabetes mellitus in 26 patients (45.6%), hypertension in 25 (43.9%), and ischemic heart disease in 7 (12.3%). Among 56 patients with calculable creatinine clearance, 20 (35.7%) had CrCl <60 mL/min. Data completeness varied across baseline variables. Smoking status was undocumented in 41 patients (71.9%), and year of diagnosis was unavailable in 36 (63.2%). Baseline creatinine was unavailable in one patient; consequently, creatinine-clearance analyses included 56 patients. Line of therapy could not be reliably determined from the available records.

Molecular profiling was incomplete. A documented del(17p) abnormality was identified in one patient, whereas no TP53 mutation was documented; however, the numbers of patients tested for these abnormalities could not be reliably ascertained. IGHV mutation status was available for only three patients, of whom two had mutated IGHV and one had unmutated IGHV. Molecular variables were therefore summarized descriptively and were not included in the adjusted toxicity models. Cytogenetic abnormalities were not mutually exclusive; trisomy 12 was the most frequent abnormality (40.4%), followed by del(13q) (29.8%) and del(11q) (19.3%). The most common starting dose was 280 mg (47.4%), followed by 420 mg (35.1%) and 140 mg (17.5%). Baseline demographic and clinical characteristics of patients treated with ibrutinib are detailed in Table 1.

**Table 1:** Baseline demographic and clinical characteristics of patients treated with ibrutinib.

Variable	Overall (*N* = 57)
Demographics	
Age, years—median (IQR)	64 (56–70)
<50, *n* (%)	7 (12.3)
50–64, *n* (%)	22 (38.6)
≥65, *n* (%)	28 (49.1)
Sex, *n* (%)	
Male	39 (68.4)
Female	18 (31.6)
Smoking status, *n* (%)	
Never	7 (12.3)
Former	7 (12.3)
Current	2 (3.5)
Not documented	41 (71.9)
BMI category, *n* (%)	
Underweight (<18.5 kg/m^2^)	1 (1.8)
Normal (18.5–24.9 kg/m^2^)	20 (35.1)
Overweight (25.0–29.9 kg/m^2^)	19 (33.3)
Obese (≥30.0 kg/m^2^)	17 (29.8)
Disease characteristics	
Diagnosis, *n* (%)	
CLL	46 (80.7)
CLL/SLL	8 (14.0)
WM	1 (1.8)
Mantle cell lymphoma	1 (1.8)
Other/unspecified B-cell malignancy	1 (1.8)
Cytogenetic abnormalities, n (%)*	
del(13q)	17 (29.8)
del(11q)	11 (19.3)
Trisomy 12	23 (40.4)
del(17p)	1 (1.8)
TP53 abnormality	0 (0.0)
IGHV mutated	2 (3.5)
IGHV unmutated	1 (1.8)
IGHV status unavailable/not documented	54 (94.7)
Normal cytogenetics	8 (14.0)
Baseline comorbidities, *n* (%)	
Hypertension (HTN)	25 (43.9)
Diabetes mellitus (DM)	26 (45.6)
Ischemic heart disease (IHD)	7 (12.3)
Chronic kidney disease	6 (10.5)
Epilepsy	2 (3.5)
Renal function (CrCl, mL/min), *n* (%)	
Normal (≥90)	23 (40.4)
Mild (60–89)	13 (22.8)
Moderate (30–59)	19 (33.3)
Severe (<30)	1 (1.8)
Missing	1 (1.8)
Treatment characteristics	
Ibrutinib starting dose, *n* (%)	
140 mg	10 (17.5)
280 mg	27 (47.4)
420 mg	20 (35.1)
Concomitant medications, *n* (%)	
Anticoagulants	2 (3.5)
Antiplatelets	5 (8.8)
Strong CYP3A4 inducers	1 (1.8)
Strong CYP3A4 inhibitors	0 (0.0)
Immunosuppressants	0 (0.0)
Vaccines	2 (3.5)

Data are presented as median (interquartile range) or number (percentage), as appropriate. BMI, body mass index; CLL, chronic lymphocytic leukemia; CrCl, creatinine clearance; CYP3A4, cytochrome P450 3A4; DM, diabetes mellitus; HTN, hypertension; IGHV, immunoglobulin heavy-chain variable region; IHD, ischemic heart disease; MCL, mantle cell lymphoma; SLL, small lymphocytic lymphoma; WM, Waldenström's macroglobulinemia. One patient lacked baseline creatinine. Cytogenetic and molecular categories were not mutually exclusive. Findings represent abnormalities documented in the available records and should not be interpreted as prevalence estimates among fully tested patients. The numbers tested for TP53 mutation and del(17p) could not be reliably determined; absence of a documented abnormality was therefore not classified as a confirmed negative result.

Overall, 37 patients (64.9%) initiated ibrutinib below the recommended 420-mg daily dose. Median age was 71 years in the 140-mg group, 61 years in the 280-mg group, and 63.5 years in the 420-mg group; the difference was not statistically significant (Kruskal–Wallis *p* = 0.105). CrCl <60 mL/min was present in 70.0%, 26.9%, and 30.0% of patients with calculable creatinine clearance in the respective groups (Fisher exact *p* = 0.060). Starting-dose category was not associated with ischemic heart disease (*p* = 0.133), Grade ≥3 adverse events (*p* = 0.583), or permanent discontinuation (*p* = 0.864). Starting-dose terms also remained nonsignificant in the reduced Firth models. Detailed dose-stratified findings are presented in Supplementary Table S3.

### 3.2 Overall adverse event profile

Because individual patients could experience multiple events, patient-level incidence and event-level frequency are reported separately. All 57 patients (100.0%) experienced at least one adverse event (AE) during ibrutinib therapy, generating a total of 220 AE events. The median number of AEs per patient was 3 (IQR 2–5; range 1–11).

The most frequently affected organ-system clusters were hematological and cardiac, each affecting 26 patients (45.6%), followed by infectious events in 24 patients (42.1%). At the event level, median time to onset varied considerably across clusters, with bleeding events occurring earliest (median 42 days) and hepatic events occurring later in the treatment course, although based on relatively few events (median 1,028 days). The complete AE cluster distribution and timing are presented in Table 2.

**Table 2:** Incidence of adverse events by organ-system cluster with timing of onset (*N* = 57 patients; 220 events).

AE cluster	Pts (*n*)	Pts (%)	Events (*n*)	Events (%)	Median time to onset, days (IQR)
Hematological	26	45.6	50	22.7	119.5 (17–339)
Cardiac	26	45.6	42	19.1	329.5 (92.8–928.2)
Infectious	24	42.1	32	14.5	147 (94.5–344.8)
Bleeding	17	29.8	25	11.4	42 (11–124)
Gastrointestinal	15	26.3	22	10.0	183 (28–370.2)
Dermatological	16	28.1	22	10.0	184 (99.8–566.5)
General	8	14.0	12	5.5	162 (68.5–280)
Hepatic	6	10.5	9	4.1	1,028 (652–1,463)
Metabolic	2	3.5	2	0.9	336 and 337^a^
Renal	2	3.5	2	0.9	35 and 42^a^
Neurological	2	3.5	2	0.9	15 and 259^a^
Any AE	57	100.0	220	100.0	157.5 (42–486.8)

Patients may appear in more than one cluster. Patient percentages are based on the total cohort (*N* = 57); event percentages are based on total events (*N* = 220). Median and IQR are calculated at the event level (cumulative ibrutinib exposure in days before AE onset).

a For clusters with fewer than five events, individual event-onset times are reported instead of a median and interquartile range. Normality was assessed by the Shapiro–Wilk test; all clusters were non-normally distributed (*p* < 0.05).

The ten most common individual AEs, each occurring in at least 10% of patients, were neutropenia (19 patients, 33.3%), hypertension (16, 28.1%), minor bleeding (16, 28.1%), upper respiratory infection (12, 21.1%), rash (12, 21.1%), thrombocytopenia (7, 12.3%), skin infection (7, 12.3%), oral mucositis (6, 10.5%), anorexia (6, 10.5%), and sinus arrhythmia (6, 10.5%) (Figure 1).

**Figure 1:**
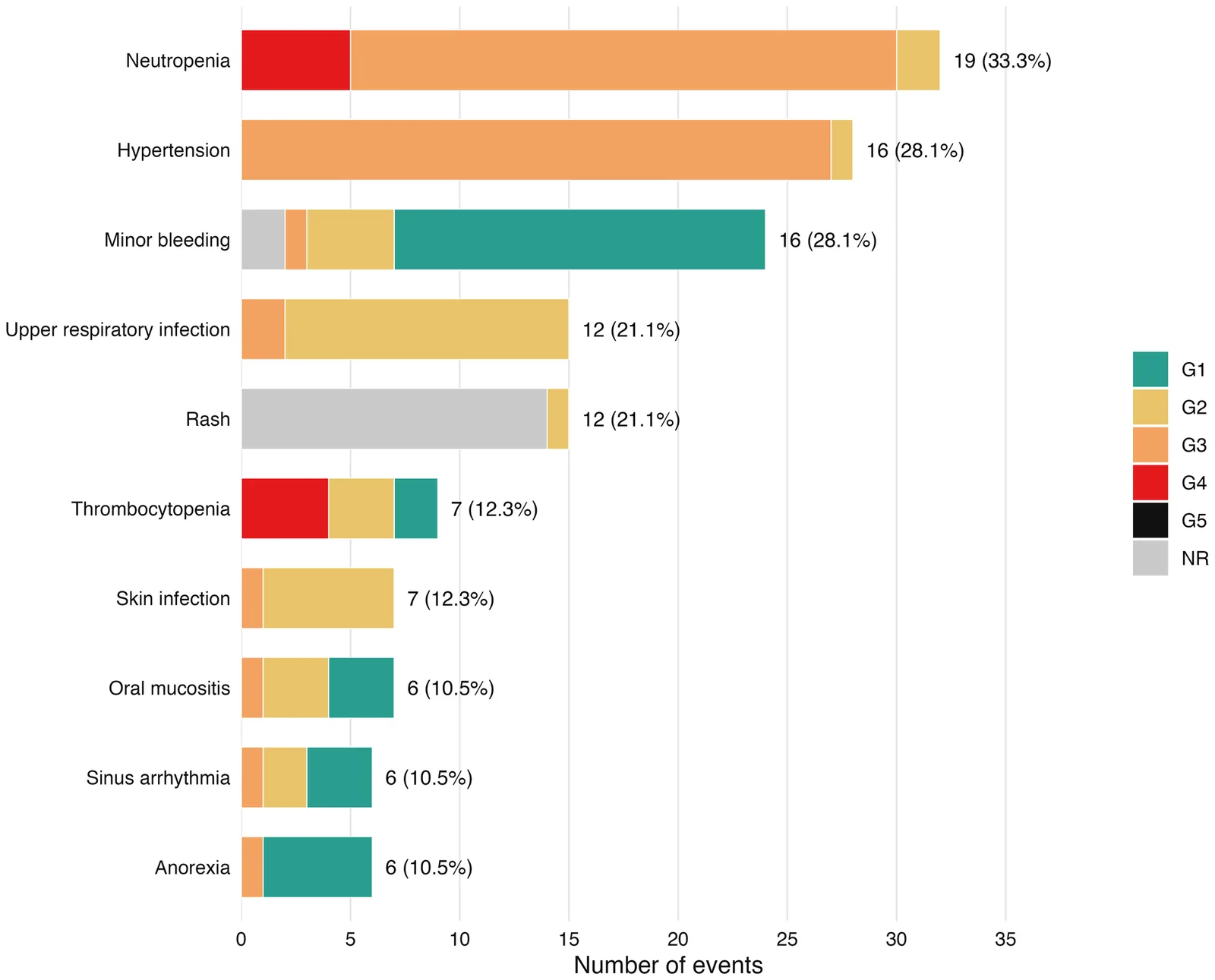
Most common adverse events (occurring in ≥10% of patients) by CTCAE v5.0 grade during ibrutinib therapy (*N* = 57). Bars represent event counts, with segments color-coded by severity grade. Labels indicate the number and percentage of patients experiencing at least one event. Grade 5 events are not shown; one fatal COVID-19 event occurred outside this subset. CTCAE, Common Terminology Criteria for Adverse Events; G1–G5, Grades 1–5; NR, grade not recorded.

### 3.3 Grade ≥3 adverse events

Of the 57 patients, 41 (71.9%) experienced at least one Grade ≥3 adverse event, accounting for 89 severe events. The highest toxicity grade was Grade 3 in 32 patients (56.1%), Grade 4 in 8 (14.0%), and Grade 5 in 1 (1.8%). Neutropenia affected 19 patients (33.3%) and hypertension affected 15 (26.3%). Hematologic and cardiac severe toxicities affected 23 (40.4%) and 19 (33.3%) patients, respectively. Median time to first Grade 3–5 adverse event was 91 days. No statistically significant differences in time to first severe adverse event were observed across baseline demographic or clinical characteristics (all log-rank *p* > 0.05) (Figure 2).

**Figure 2:**
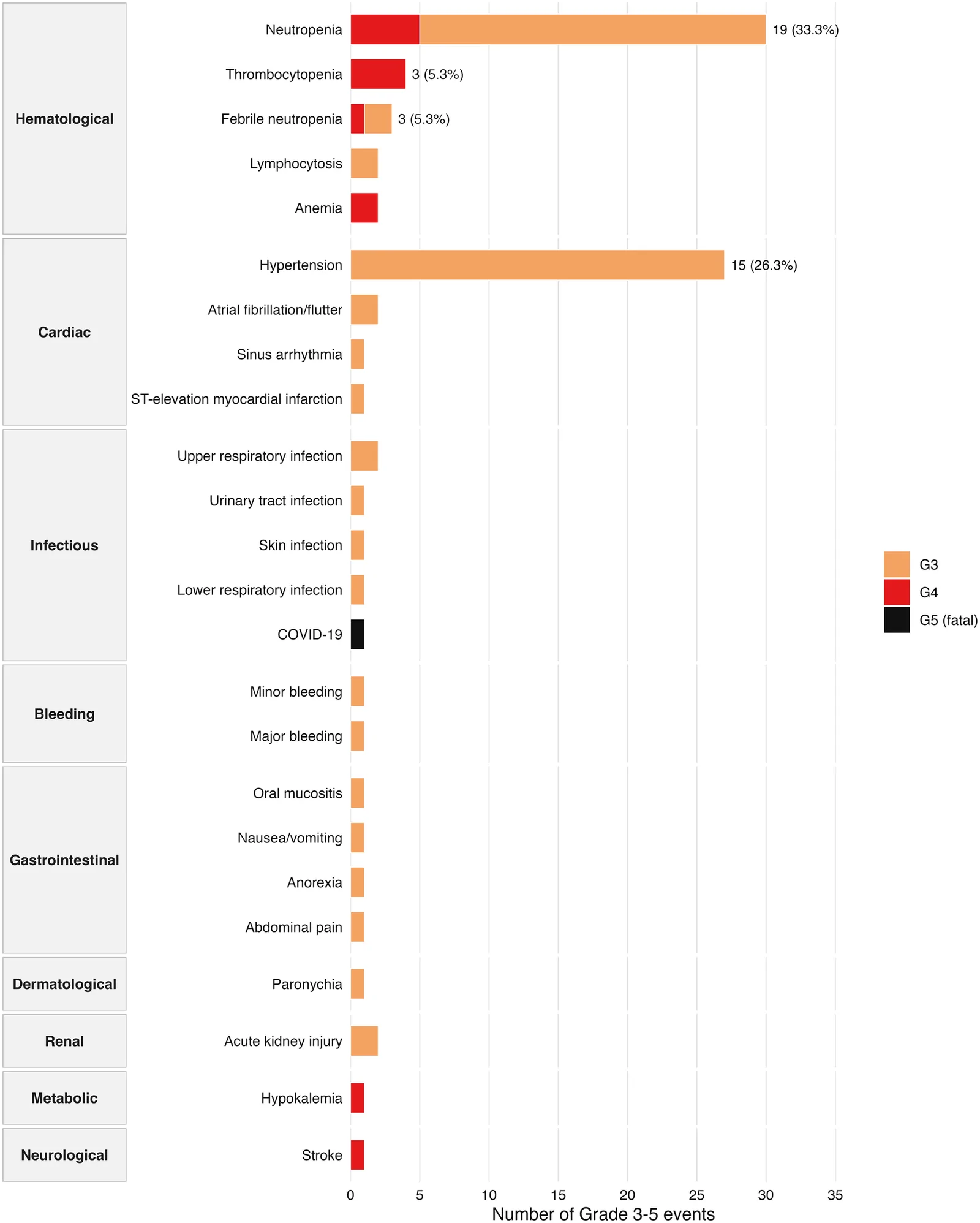
Grade 3–5 adverse events by individual type and organ-system cluster during ibrutinib therapy (*N* = 57 patients; 89 events). Bars represent event counts stratified by CTCAE v5.0 severity: G3 (severe), G4 (life-threatening), and G5 (fatal). Labels indicate patient-level incidence for adverse events occurring in ≥5% of patients. Organ-system clusters are displayed in separate panels. Hepatic and general clusters are not shown because no Grade 3–5 events occurred in these clusters.

### 3.4 Dose modifications and discontinuation

Forty-seven patients (82.5%) required at least one dose modification during ibrutinib therapy, whereas 10 (17.5%) experienced adverse events that did not result in a documented treatment modification. Dose interruption occurred in 36 patients (63.2%), dose reduction in 27 (47.4%), and permanent discontinuation due to adverse events in 17 (29.8%). Categories were not mutually exclusive (Table 3).

**Table 3:** Dose modifications during ibrutinib therapy.

Dose modification	*n*/57	%
Any dose modification	47/57	82.5
Dose interruption (hold)	36/57	63.2
Dose reduction	27/57	47.4
Permanent discontinuation	17/57	29.8

Data are presented as patient-level counts; each patient is counted once per category regardless of the number of events.

Among the 17 patients who permanently discontinued ibrutinib, the majority (10, 58.8%) had their AEs resolve. Three patients (17.6%) died: one from COVID-19 infection (Grade 5, day 418), one from concurrent upper respiratory infection and acute kidney injury (Grade 3, day 42), and one from stroke (Grade 4, day 15). Two patients (11.8%) were switched to a second-generation BTK inhibitor. Cardiac, bleeding, and dermatologic adverse events were the most frequent clusters associated with discontinuation, each affecting four patients.

### 3.5 Risk factor analysis

Given the limited number of outcome events, adjusted analyses were repeated using reduced Firth penalized-likelihood logistic regression models. In the permanent-discontinuation model, pre-existing IHD was associated with discontinuation after adjustment for starting-dose category (aOR 6.25, 95% profile-likelihood CI 1.29–39.19; *p* = 0.023). Starting dose was not significantly associated with discontinuation (280 vs. 420 mg: aOR 0.91, 95% CI 0.25–3.52; *p* = 0.891; 140 vs. 420 mg: aOR 0.82, 95% CI 0.14–4.21; *p* = 0.812). The apparent C-index was 0.649 (95% CI 0.495–0.804) (Figure 3A).

**Figure 3:**
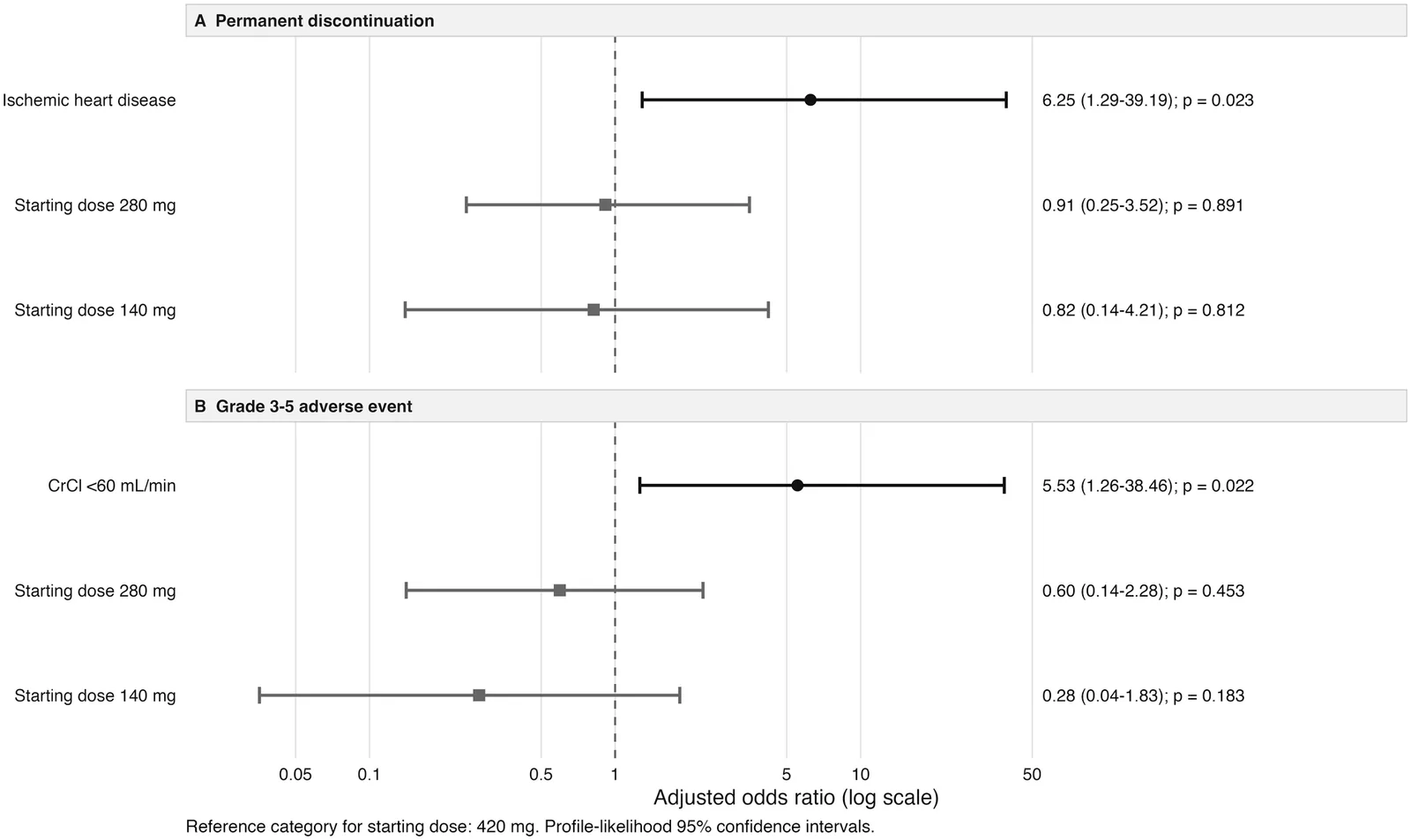
Reduced firth penalized-likelihood logistic regression models for **(A)** permanent ibrutinib discontinuation and **(B)** grade 3–5 adverse events in the final cohort (*N* = 57). Models included the prespecified clinical predictor and starting-dose category. Points represent adjusted odds ratios (aORs), and horizontal lines represent profile-likelihood 95% confidence intervals. The dashed vertical line indicates an aOR of 1.0. The reference starting dose was 420 mg. CrCl, creatinine clearance; IHD, ischemic heart disease.

Among 56 patients with calculable creatinine clearance, CrCl <60 mL/min was associated with Grade 3–5 toxicity after adjustment for starting-dose category (aOR 5.53, 95% profile-likelihood CI 1.26–38.46; *p* = 0.022). Starting dose was not significant (280 vs. 420 mg: aOR 0.60, 95% CI 0.14–2.28; *p* = 0.453; 140 vs. 420 mg: aOR 0.28, 95% CI 0.04–1.83; *p* = 0.183). The apparent C-index was 0.724 (95% CI 0.576–0.871) (Figure 3B).

In the CLL/SLL-only cohort (*N* = 54), all patients experienced at least one AE, 39 (72.2%) experienced Grade 3–5 toxicity, and 16 (29.6%) permanently discontinued treatment because of toxicity. The associations remained similar for IHD and discontinuation (aOR 5.86, 95% CI 1.20–36.80; *p* = 0.029) and for CrCl <60 mL/min and severe toxicity (aOR 5.27, 95% CI 1.17–37.34; *p* = 0.029).

One excluded patient switched from ibrutinib to acalabrutinib after approximately 14 days, with no adverse event documented during ibrutinib exposure. When this record was included as an additional patient with no documented adverse event, the incidence of any adverse event was 57/58 (98.3%), Grade ≥3 toxicity was 41/58 (70.7%), and permanent discontinuation due to an adverse event was 17/58 (29.3%). The corresponding estimates in the primary cohort were 57/57 (100.0%), 41/57 (71.9%), and 17/57 (29.8%). Inclusion of this record did not materially alter the descriptive findings; however, undocumented early toxicity or a toxicity-related reason for switching cannot be excluded.

### 3.6 Time-to-event analysis

Kaplan–Meier analysis demonstrated significantly earlier treatment discontinuation among patients with pre-existing IHD compared with those without (log-rank *p*=.016). Median time to discontinuation was 225 days in the IHD group and was not reached in patients without IHD. The Cox proportional-hazards estimate was 3.38 (95% CI 1.18–9.65; *p* = 0.023), and the log-rank *p*-value was 0.016 (Figure 4).

**Figure 4:**
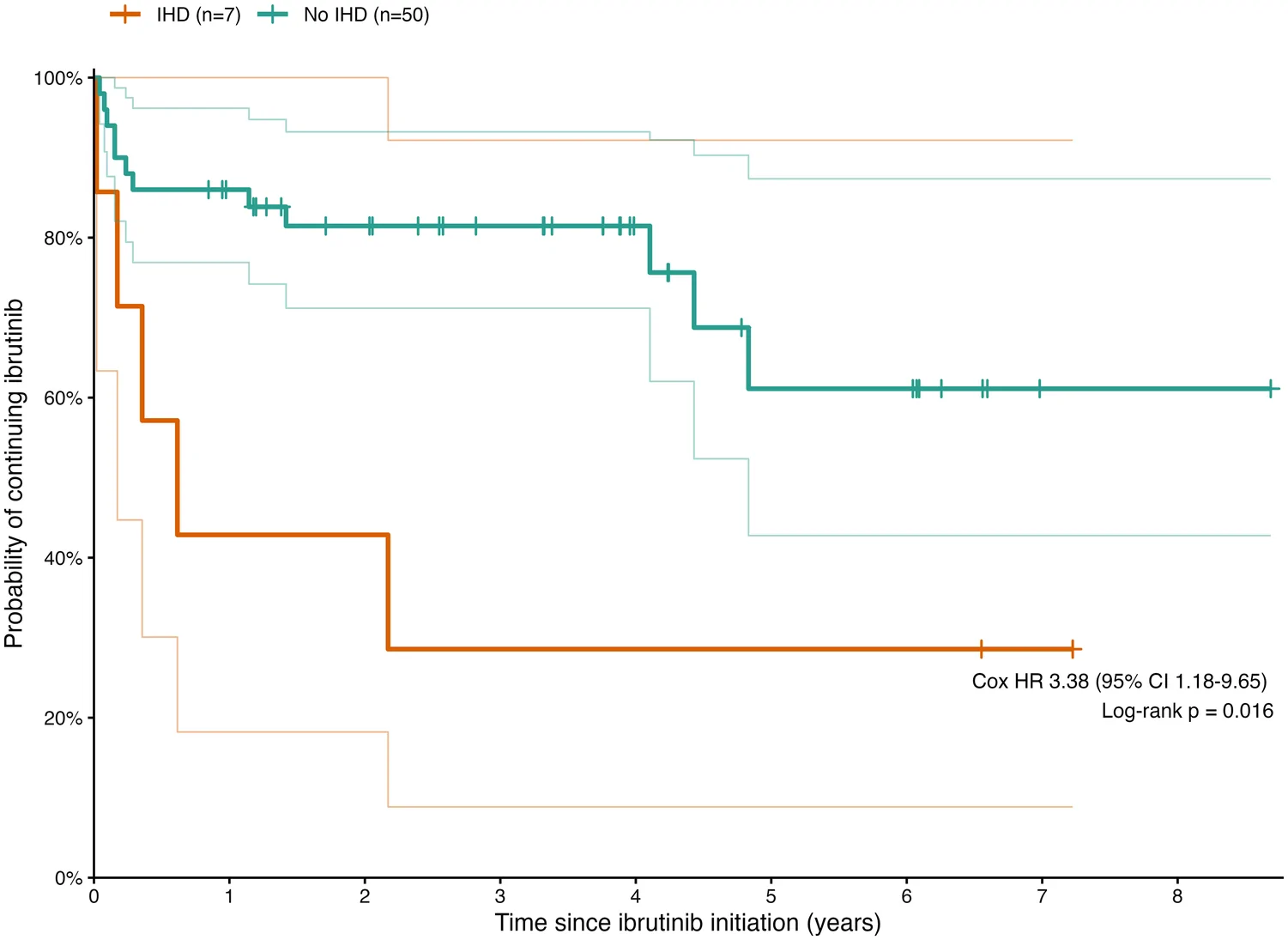
Kaplan–Meier estimates of the probability of continuing ibrutinib according to baseline ischemic heart disease status. Tick marks indicate censored observations, and thin lines represent 95% confidence limits. Patients with IHD (*n* = 7) experienced earlier permanent discontinuation than patients without IHD (*n* = 50; log-rank *p* = 0.016). The Cox proportional-hazards estimate was HR 3.38 (95% CI 1.18–9.65). Median time to discontinuation was 225 days in the IHD group and was not reached in the group without IHD.

### 3.7 Temporal pattern of adverse events

At the patient level, the omnibus paired permutation test indicated a difference in adverse-event cluster profiles between the early period and the full period after day 90 (permutation statistic 24.288; *p* = 0.0046). Cardiac events occurred in 9 patients (15.8%) during the early period and 20 (35.1%) during the late period, while infectious events occurred in 8 (14.0%) and 19 (33.3%), respectively. Hematologic events occurred in 13 (22.8%) and 16 (28.1%) patients, and bleeding events in 11 (19.3%) and 8 (14.0%), respectively. None of the 11 cluster-specific exact McNemar comparisons remained statistically significant after Holm correction. In the equal-window sensitivity analysis comparing days 0–90 with days 91–180 among 47 evaluable patients, the global paired difference was not significant (permutation statistic 9.855; *p* = 0.240), and no cluster-specific comparison was significant (Figure 5).

**Figure 5:**
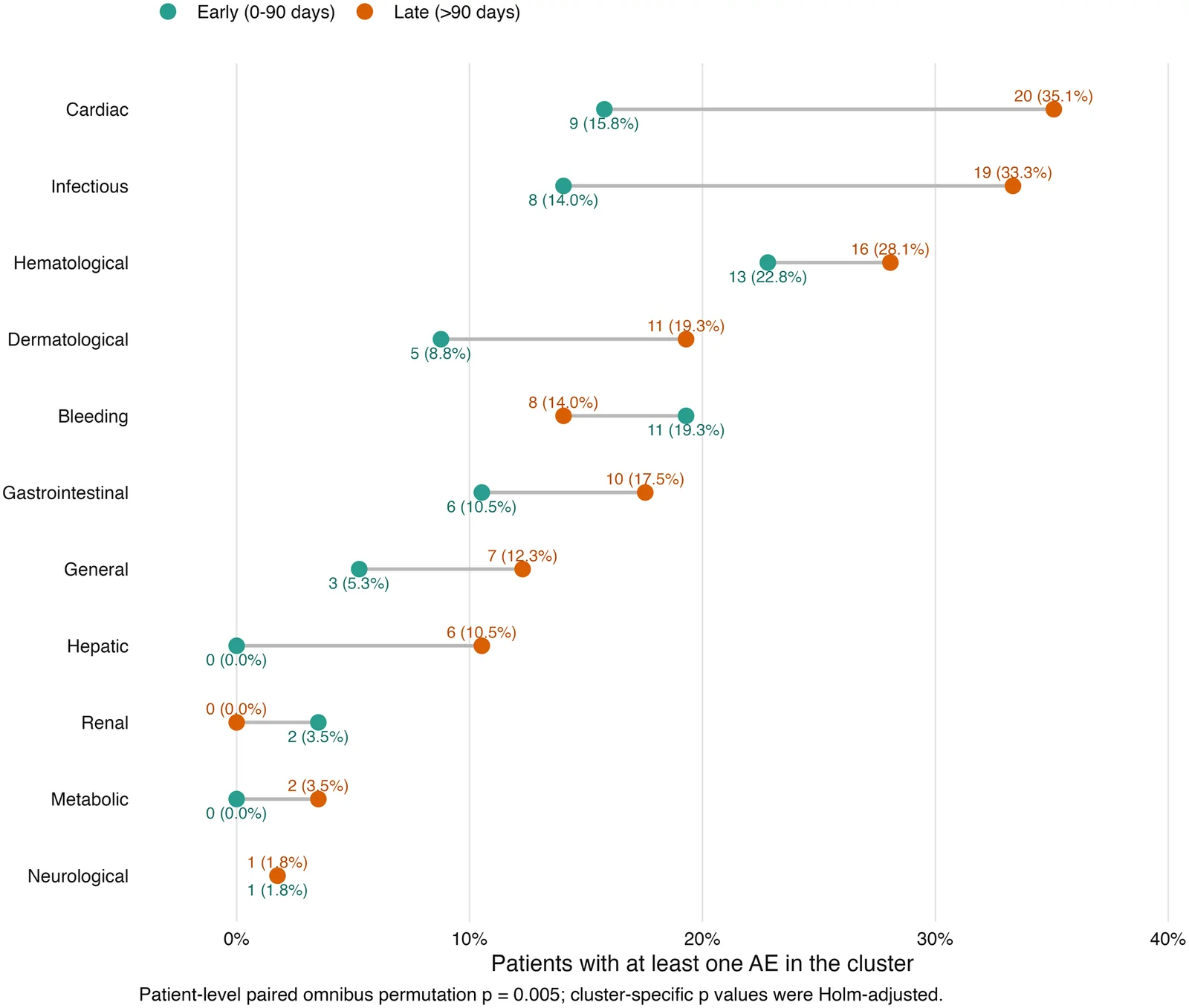
Patient-level occurrence of adverse events by organ-system cluster during the early (0–90 days) and late (>90 days) treatment periods (*N* = 57). Each percentage represents the proportion of patients experiencing at least one adverse event in the specified cluster and period. The overall paired cluster distribution differed between periods in the omnibus permutation analysis (*p* = 0.00460; 100,000 permutations). Cluster-specific comparisons used exact McNemar tests with Holm correction; none remained statistically significant after multiplicity adjustment.

### 3.8 Adverse event burden

Patients with Grade ≥3 toxicity had a median of 3 adverse events per patient (IQR 3–5), compared with 2.5 (IQR 1.75–4) among patients without severe toxicity; this difference was not statistically significant (Mann–Whitney *p* = 0.064; Figure 6). Total adverse-event burden was not associated with permanent discontinuation (*p* = 0.811). Patients with diabetes had lower total adverse-event counts than those without diabetes (median 3, IQR 2–3.75, vs. 4, IQR 3–6; *p* = 0.008).

**Figure 6:**
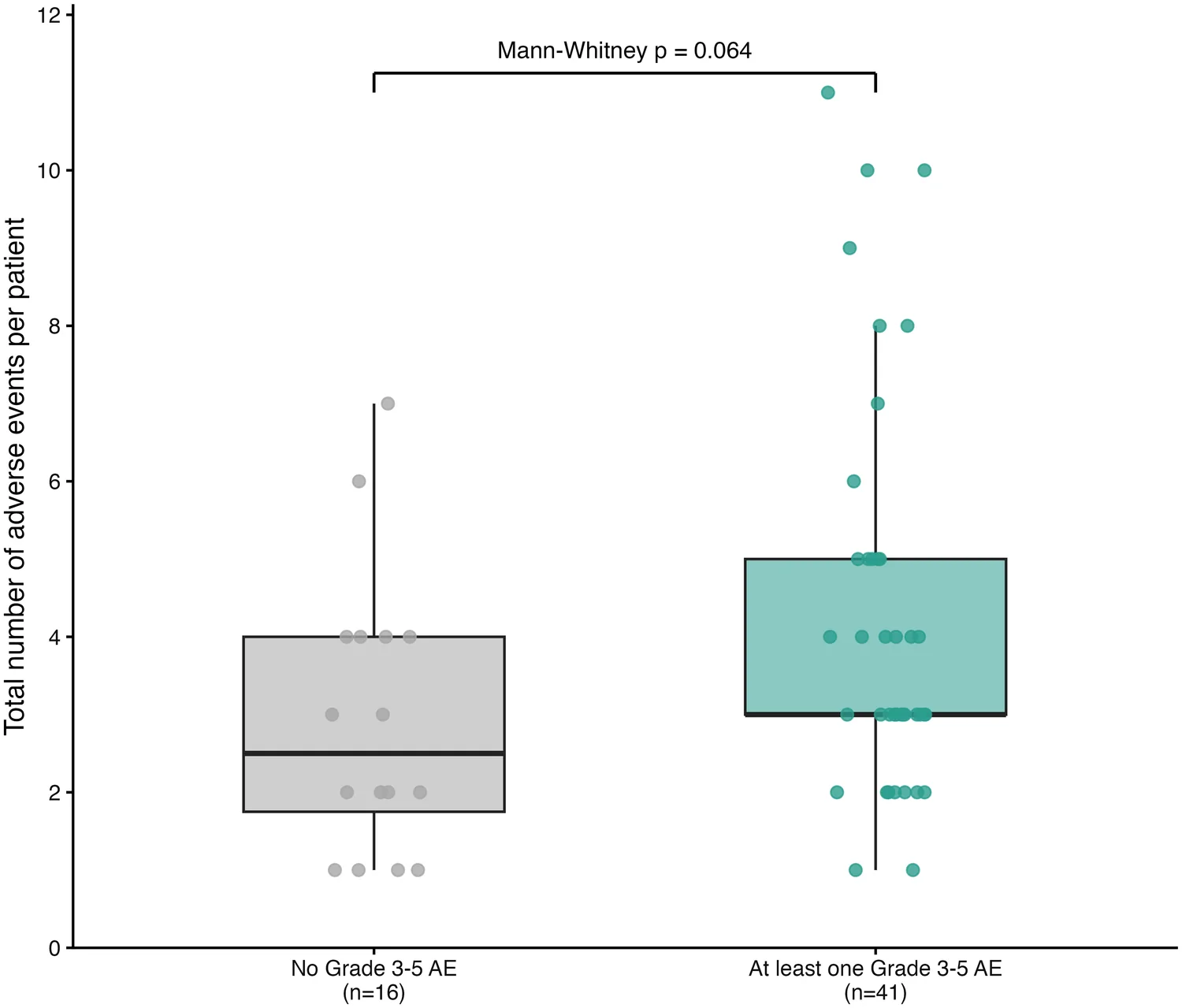
Total adverse-event count per patient according to grade 3–5 toxicity status in the final cohort (*N* = 57). Boxes represent the interquartile range, horizontal lines represent medians, whiskers extend to the most extreme observations within 1.5 times the interquartile range, and points represent individual patients. Total AE burden did not differ significantly between patients with and without Grade 3–5 toxicity (Mann–Whitney *p* = 0.064).

### 3.9 Hold duration

Thirty-six patients (63.2%) experienced at least one treatment hold. After records with identical hold and resumption dates were treated as the same clinical episode, 74 unique hold episodes were identified. Duration was documented for 61 episodes among 33 patients, with a median duration of 7 days (IQR 7–21; range 2–176). Using each patient's median documented hold duration, hold duration did not differ according to Grade ≥3 toxicity status (Mann–Whitney *p* = 0.772) or permanent discontinuation status (*p* = 0.596).

### 3.10 Dose reduction patterns

Dose reduction occurred in 27 patients (47.4%), comprising 43 documented dose-reduction events. The most frequent reduction patterns were from 420 to 280 mg (21/43, 48.8%) and from 280 to 140 mg (20/43, 46.5%); reduction from 420 to 140 mg occurred in two instances (4.7%). One additional record documented an increase from 280 to 420 mg and was therefore classified as dose re-escalation rather than dose reduction. The clinical rationale for this re-escalation could not be verified. Starting-dose category was not associated with the likelihood of subsequent dose reduction (*p* = 0.437). Twenty of the 27 patients who underwent dose reduction (74.1%) experienced a subsequent adverse event.

### 3.11 Calendar-era sensitivity analysis

Treatment-initiation dates were available for all 57 patients. Twenty-four initiated ibrutinib before 2022 and 33 initiated during 2022–2025. Grade ≥3 adverse events occurred in 20/24 patients (83.3%) in the earlier group and 21/33 (63.6%) in the later group (Fisher exact *p* = 0.139). Permanent discontinuation occurred in 11/24 (45.8%) and 6/33 (18.2%), respectively (*p* = 0.039). Initiation below 420 mg occurred in 12/24 patients (50.0%) in the earlier group and 25/33 (75.8%) in the later group (*p* = 0.054).

Median potential administrative follow-up was 2,295.5 days for patients initiating before 2022 and 874 days for those initiating during 2022–2025. Later initiation was not associated with permanent discontinuation in time-to-event analysis (HR 0.54, 95% CI 0.19–1.55; Cox *p* = 0.251; log-rank *p* = 0.246) or with time to first Grade ≥3 adverse event (HR 0.75, 95% CI 0.41–1.39; Cox *p* = 0.364; log-rank *p* = 0.363). Complete comparisons are presented in Supplementary Table S4.

## 4 Discussion

This real-world study demonstrates that ibrutinib-associated toxicity remains clinically consequential in routine practice, frequently requiring treatment interruption, dose modification, or permanent discontinuation. Beyond describing the toxicity profile, the study identifies exploratory associations between baseline cardiovascular and renal vulnerability and treatment outcomes, while the temporal analysis illustrates the methodological importance of accounting for repeated events and unequal observation periods. Collectively, the findings emphasize the complexity of maintaining long-term ibrutinib therapy in a clinically heterogeneous population.

### 4.1 Comparison with international ibrutinib safety data

The overall burden of severe toxicity was broadly consistent with that reported in long-term clinical trials and observational cohorts, although the distribution of individual toxicities differed (3, 4, 8, 9). Compared with pivotal trials, atrial fibrillation and major bleeding appeared less prominent, whereas hypertension and neutropenia were more frequently represented. Similar variation has been observed across real-world studies and probably reflects differences in age, comorbidity burden, disease characteristics, follow-up duration, supportive care, and adverse-event ascertainment.

Discontinuation appeared more consistent with real-world experience than with controlled trials. Clinical-trial participants are typically more selectively enrolled and followed under protocolized monitoring, whereas routine-care populations include patients with greater physiological vulnerability, polypharmacy, and competing comorbidities. Differences between clinical-trial and observational discontinuation rates should therefore not be interpreted as direct comparisons of treatment safety. The international comparison in Supplementary Table S1 provides context but does not eliminate heterogeneity between studies.

### 4.2 Ischemic heart disease and treatment discontinuation

Pre-existing ischemic heart disease was associated with permanent ibrutinib discontinuation in both penalized regression and time-to-event analyses. Cardiovascular toxicities of ibrutinib, particularly hypertension and atrial fibrillation, are well established, but the specific contribution of pre-existing coronary disease to treatment intolerance is less clearly defined (16–18).

Several explanations are possible. Patients with established ischemic heart disease may have reduced cardiovascular reserve and greater susceptibility to the hemodynamic or electrophysiological effects of therapy. Ibrutinib-related effects on platelet signaling and off-target cardiovascular pathways may also be less well tolerated in this population (19, 20). Alternatively, clinicians may have adopted a lower threshold for interruption or discontinuation when treating patients with known cardiovascular disease, even when the precipitating adverse event was not directly ischemic.

The association should nevertheless be regarded as exploratory. The ischemic-heart-disease subgroup was small, the confidence intervals were wide, and residual confounding by cardiovascular severity, frailty, concomitant medications, or clinician decision-making cannot be excluded. The findings therefore support greater awareness of baseline cardiovascular vulnerability but do not establish ischemic heart disease as a validated predictor or as an independent basis for selecting a specific BTK inhibitor.

### 4.3 Renal impairment and severe toxicity

Impaired renal function was associated with Grade ≥3 toxicity after adjustment for starting-dose category. Because ibrutinib is predominantly metabolized hepatically, this association should not be interpreted as evidence of impaired renal elimination of the drug. Renal impairment may instead serve as a marker of broader physiological vulnerability, including multimorbidity, anemia, systemic inflammation, altered handling of concomitant medications, and increased susceptibility to infectious or hematologic complications.

This finding also remains exploratory because of the limited cohort size and imprecision of the effect estimate. Baseline renal function may contribute to an overall assessment of treatment vulnerability and monitoring requirements, but the present data do not establish a renal-function threshold that independently mandates ibrutinib dose reduction.

### 4.4 Temporal patterns of adverse events

The patient-level temporal analysis suggested that the overall distribution of adverse-event clusters differed when the initial treatment period was compared with the entire subsequent follow-up. Cardiac and infectious events appeared more prominent during later follow-up, whereas bleeding events were relatively more concentrated near treatment initiation. Such patterns are clinically plausible because platelet-related effects may manifest early, while cardiovascular and infectious complications may emerge during prolonged exposure.

However, no individual adverse-event cluster remained statistically significant after correction for multiple comparisons. In addition, the equal-window sensitivity analysis did not demonstrate a significant temporal difference. The apparent late predominance of some toxicities may therefore partly reflect the longer opportunity for events to accumulate after the initial treatment period.

These findings do not support a formal phase-specific monitoring schedule. Rather, they reinforce the need for continued surveillance throughout therapy and demonstrate that conclusions about adverse-event timing depend on the duration and structure of the comparison periods. Larger longitudinal studies using recurrent-event methods are needed to characterize temporal toxicity trajectories more reliably.

### 4.5 Starting-dose selection and calendar-time effects

Most patients initiated ibrutinib below the standard CLL/SLL dose of 420 mg. Reduced-dose initiation has also been described in other real-world cohorts, where clinician preference, comorbidities, concomitant medications, and anticipated tolerability influenced prescribing (21). In the present cohort, patients receiving the lowest starting dose appeared clinically more vulnerable, but the reasons for individual dose selection were not consistently documented.

Starting-dose category was not associated with severe toxicity or discontinuation in the adjusted models. Nevertheless, this should not be interpreted as evidence that starting doses are interchangeable. Dose selection was clinician directed rather than randomized and may have incorporated frailty, comorbidity severity, drug interactions, or other factors unavailable in the dataset. Confounding by indication therefore remains likely, and the study cannot determine whether reduced-dose initiation improves tolerability or preserves treatment effectiveness.

The calendar-era analysis similarly requires cautious interpretation. Later initiators had shorter potential follow-up and differed in clinical characteristics and prescribing patterns. The absence of a clear time-to-event association provides some reassurance that calendar period alone did not explain the primary findings, but evolving institutional practices and changes in patient selection cannot be excluded.

### 4.6 Dose modification and persistent toxicity

Treatment interruptions and reductions were common, illustrating the practical difficulty of maintaining continuous therapy. Many patients experienced further adverse events after dose reduction, but this does not establish that dose reduction was ineffective. Patients requiring reduction had already demonstrated treatment intolerance and may have been intrinsically more susceptible to additional toxicity. Continued follow-up after reduction also created further opportunity for adverse events to be documented.

The analysis therefore cannot determine the causal effect of dose reduction on subsequent toxicity. Persistent or recurrent adverse events should prompt reassessment of comorbidities, interacting medications, treatment goals, and the overall suitability of continued ibrutinib therapy. Consideration of alternative BTK inhibitors may be reasonable when clinically appropriate, but comparative treatment recommendations cannot be derived from this uncontrolled cohort.

### 4.7 Cumulative adverse-event burden

Cumulative adverse-event burden was not significantly associated with Grade ≥3 toxicity after the final cohort correction. The modest difference between groups may reflect broader treatment vulnerability, but the analysis does not demonstrate that accumulating lower-grade events predict subsequent severe toxicity. Furthermore, adverse-event burden was measured over the complete treatment course, preventing determination of whether lower-grade events preceded or followed severe events.

Cumulative burden should therefore be presented as a descriptive measure rather than a warning marker or risk-prediction tool. Prospective analyses that define an early assessment window and evaluate subsequent severe toxicity would be required to test its predictive value.

## 5 Strengths and limitations

The study has several strengths. Adverse events were graded using CTCAE v5.0, and the analysis incorporated patient-level incidence, event characteristics, treatment modification, survival outcomes, and sensitivity analyses. The use of reduced Firth penalized models was appropriate for addressing sparse data, while the patient-level temporal reanalysis directly addressed the dependence of repeated adverse events. The study also contributes safety evidence from a Middle Eastern population that remains underrepresented in the international literature.

The findings must nevertheless be interpreted within the constraints of a retrospective, single-center design. Adverse-event detection depended on routine documentation, and lower-grade events may have been incompletely recorded. Excluded records lacked sufficient baseline information for comparison with included patients, so selection bias may have affected toxicity estimates in either direction. Treatment line, frailty, performance status, reasons for reduced-dose initiation, and some molecular characteristics were unavailable or incompletely documented. Missing or incomplete documentation limited adjustment for potentially relevant factors. In particular, smoking status was undocumented in most patients, while year of diagnosis and line of therapy could not be reliably determined. Molecular testing was also incomplete. These limitations precluded adjustment for smoking, treatment line, diagnosis-year effects, and selected molecular characteristics and may have contributed to residual confounding. Although year of diagnosis was frequently unavailable, treatment-initiation dates were available for all patients and were used in the calendar-era sensitivity analysis.

The adjusted analyses were limited by the small cohort and limited outcome counts. Firth penalization reduces small-sample and sparse-data bias but cannot create information absent from the dataset. The wide confidence intervals and modest apparent discrimination underscore the exploratory nature of the associations. Model performance was evaluated in the derivation cohort without internal optimism correction or external validation.

The cohort was predominantly composed of patients with CLL/SLL, and the sensitivity analysis supports interpretation primarily within that population. The lack of an active comparator prevents conclusions about relative safety vs. second-generation BTK inhibitors. Finally, the temporal findings were sensitive to the selected comparison windows, and the cumulative-burden analysis did not establish temporal prediction. Confirmation in larger multicenter cohorts with standardized prospective adverse-event collection is required.

## 6 Conclusion

Ibrutinib toxicity remained clinically important in routine practice and frequently affected treatment continuity. Pre-existing ischemic heart disease and impaired renal function emerged as exploratory markers of treatment vulnerability, but neither should be regarded as a validated predictor. The temporal findings support continued surveillance throughout therapy but do not establish a phase-specific monitoring schedule. These results emphasize individualized assessment of comorbidity, concomitant therapy, and emerging toxicity while supporting prospective validation in larger, clinically characterized cohorts.

## Data Availability

The data analyzed in this study is subject to the following licenses/restrictions: data set contains patients clinical information. Requests to access these datasets should be directed to Maan H. Harbi mhiharbi@uqu.edu.sa.
