# Supplementary material for: Real-world toxicity and discontinuation risk in ibrutinib therapy: a retrospective cohort study
Source: Front Med (Lausanne). 2026 Sep 14;13:1897420. doi: 10.3389/fmed.2026.1897420 (PMC13616786; doi:10.3389/fmed.2026.1897420)
Supplement: Supplementary file 1 [file Table1.docx]

**Supplementary Tables S1-S5**

Real-World Toxicity and Discontinuation Risk in Ibrutinib Therapy: A Retrospective Cohort Study | Final analytic cohort: N=57

**Supplementary Table S1. Descriptive international context for reported ibrutinib safety outcomes**

The present N=57 cohort is shown alongside selected pivotal and real-world studies cited in the manuscript.

| **Feature** | **Present cohort** | **RESONATE-2, 5-year follow-up** | **FIRE, CLL/SLL safety cohort** | **Romanian real-world cohort** | **Coutre integrated safety analysis** |
| --- | --- | --- | --- | --- | --- |
| Design / population | Retrospective, single-center; B-cell malignancies | Phase 3; previously untreated CLL/SLL, age >=65 years | Prospective real-world; CLL/SLL, predominantly relapsed/refractory | Retrospective, single-center; CLL/SLL, predominantly relapsed/refractory | Integrated phase 3 trials; treatment-naive and relapsed/refractory CLL/SLL |
| Safety population | 57 | 135 | 202 | 123 | 330 |
| Reported follow-up or exposure | Variable real-world exposure; data cutoff December 2025 | Median follow-up 60 months | Median follow-up 17.7 months | Median follow-up 32 months | Median exposure 29.0 months (range 0.2-42.9) |
| Any AE, n (%) | 57 (100.0) | NR | 197 (97.5) | 101 (82.1) | 330 (100.0) |
| Grade >=3 or severe AE, n (%) | 41 (71.9), Grade 3-5 | 112 (83.0), Grade >=3 | 113 (55.9), severe TEAE | 38 (30.9), Grade >=3 | Overall rate NR |
| Atrial fibrillation/flutter | Any grade: 4 (7.0); Grade 3-5: 2 (3.5) | Any grade: 16% | Any grade: 12 (5.9) | Any grade: 11 (8.9) | Any grade: 36 (10.9) |
| Hypertension | Any grade: 16 (28.1); Grade 3-5: 15 (26.3) | Grade >=3: 8% | Any grade: 21 (10.4) | Any grade: 29 (23.6) | Any grade: 69 (20.9); Grade 3: 21 (6.4) |
| Bleeding outcome* | Any bleeding: 17 (29.8); major: 1 (1.8) | Major hemorrhage: 11% | Any bleeding: 88 (43.6); major: 10 (5.0) | Bruising: 30 (24.4); major bleeding NR | Any bleeding: 182 (55.2); Grade >=3: 17 (5.2) |
| Neutropenia | Any grade: 19 (33.3); Grade 3-5: 19 (33.3) | Grade >=3: 13% | NR in main report | Any grade: 26 (21.1) | Grade 3/4: 60 (18.2) |
| Discontinuation due to AE/toxicity | 17 (29.8) | 38 (28.1) | 26 (12.9) | 11 (8.9) | 37 (11.2) |

*Bleeding definitions were not uniform: reported outcomes included any bleeding, major bleeding/major hemorrhage, bruising, or Grade >=3 bleeding. AE ascertainment, severity definitions, CTCAE versions, follow-up, and patient populations differed substantially across studies; therefore, this table is descriptive and should not be interpreted as a direct comparative safety analysis. NR, not reported; TEAE, treatment-emergent adverse event.

Published comparator data: Burger et al., Leukemia 2020 (RESONATE-2); Dartigeas et al., Clin Hematol Int 2022 (FIRE); Moldovianu et al., Medicina 2023; and Coutre et al., Blood Advances 2019.

**Supplementary Table S2. Disease-mix sensitivity analysis restricted to CLL/SLL**

Primary outcomes and reduced Firth penalized-likelihood models in the full cohort and the CLL/SLL-only cohort.

| **Outcome or model term** | **Full cohort (N=57)** | **CLL/SLL only (N=54)** |
| --- | --- | --- |
| **Descriptive safety outcomes** | | |
| Any adverse event, n (%) | 57 (100.0) | 54 (100.0) |
| Grade 3-5 adverse event, n (%) | 41 (71.9) | 39 (72.2) |
| Permanent discontinuation due to an AE, n (%) | 17 (29.8) | 16 (29.6) |
| **Reduced Firth model: permanent discontinuation** | | |
| Ischemic heart disease, aOR (95% CI); p | 6.25 (1.29-39.19); p = 0.023 | 5.86 (1.20-36.80); p = 0.029 |
| Starting dose 280 vs 420 mg, aOR (95% CI); p | 0.91 (0.25-3.52); p = 0.891 | 0.68 (0.17-2.72); p = 0.575 |
| Starting dose 140 vs 420 mg, aOR (95% CI); p | 0.82 (0.14-4.21); p = 0.812 | 0.72 (0.12-3.72); p = 0.692 |
| Apparent C-index (95% DeLong CI) | 0.649 (0.495-0.804) | 0.641 (0.469-0.814) |
| **Reduced Firth model: Grade 3-5 adverse events** | | |
| CrCl <60 mL/min, aOR (95% CI); p | 5.53 (1.26-38.46); p = 0.022 | 5.27 (1.17-37.34); p = 0.029 |
| Starting dose 280 vs 420 mg, aOR (95% CI); p | 0.60 (0.14-2.28); p = 0.453 | 0.44 (0.09-1.80); p = 0.258 |
| Starting dose 140 vs 420 mg, aOR (95% CI); p | 0.28 (0.04-1.83); p = 0.183 | 0.23 (0.03-1.59); p = 0.135 |
| Apparent C-index (95% DeLong CI) | 0.724 (0.576-0.871) | 0.736 (0.582-0.891) |

The CLL/SLL-only cohort included 46 patients coded as CLL and 8 coded as CLL/SLL. It excluded one patient with Waldenstrom's macroglobulinemia, one with mantle cell lymphoma, and one with another/unspecified B-cell malignancy. Both reduced Firth models included starting-dose category, with 420 mg as the reference. Regression confidence intervals are profile-likelihood 95% CIs. C-indices are apparent (in-sample) discrimination estimates and were not externally validated. AE, adverse event; aOR, adjusted odds ratio; CI, confidence interval; CLL, chronic lymphocytic leukemia; CrCl, Cockcroft-Gault creatinine clearance; SLL, small lymphocytic lymphoma.

**Supplementary Table S3. Baseline characteristics and safety outcomes according to ibrutinib starting dose**

Exploratory assessment of potential confounding associated with clinician-selected starting-dose category in the final N=57 cohort.

**A. Dose-stratified descriptive characteristics and outcomes**

| **Characteristic or outcome** | **420 mg (N=20)** | **280 mg (N=27)** | **140 mg (N=10)** | **Overall p** |
| --- | --- | --- | --- | --- |
| Age, years, median (IQR) | 63.5 (52.25-68.25) | 61 (55-69) | 71 (63.5-74.5) | 0.105 |
| Pre-existing ischemic heart disease, n (%) | 4 (20.0) | 1 (3.7) | 2 (20.0) | 0.133 |
| CrCl <60 mL/min, n/N available (%) | 6/20 (30.0) | 7/26 (26.9) | 7/10 (70.0) | 0.060 |
| Grade 3-5 adverse event, n (%) | 16 (80.0) | 18 (66.7) | 7 (70.0) | 0.583 |
| Permanent discontinuation due to an AE, n (%) | 7 (35.0) | 7 (25.9) | 3 (30.0) | 0.864 |

**B. Starting-dose terms in the reduced Firth penalized-likelihood models**

| **Outcome model** | **420 mg** | **280 mg vs 420 mg** | **140 mg vs 420 mg** |
| --- | --- | --- | --- |
| Permanent discontinuation | Reference | 0.91 (0.25-3.52); p = 0.891 | 0.82 (0.14-4.21); p = 0.812 |
| Grade 3-5 adverse events | Reference | 0.60 (0.14-2.28); p = 0.453 | 0.28 (0.04-1.83); p = 0.183 |

Panel A presents median (IQR), n (%), or n/N available (%). Age was compared using the Kruskal-Wallis test; categorical variables were compared using Fisher's exact test. Panel B reports adjusted odds ratios with profile-likelihood 95% CIs and term-specific penalized likelihood-ratio p-values. The discontinuation model included ischemic heart disease and starting-dose category; the Grade 3-5 model included CrCl <60 mL/min and starting-dose category. The patient-level clinical rationale for reduced-dose initiation was not consistently documented. AE, adverse event; CI, confidence interval; CrCl, Cockcroft-Gault creatinine clearance; IQR, interquartile range.

**Supplementary Table S4. Calendar-era sensitivity analysis according to ibrutinib treatment-initiation period**

Comparison of patients initiating ibrutinib before 2022 with those initiating during 2022-2025 in the final N=57 cohort.

**A. Descriptive calendar-era comparison**

| **Characteristic or outcome** | **Before 2022 (N=24)** | **2022-2025 (N=33)** | **Overall p** |
| --- | --- | --- | --- |
| Pre-existing ischemic heart disease, n (%) | 5 (20.8) | 2 (6.1) | 0.119 |
| CrCl <60 mL/min, n/N available (%) | 9/23 (39.1) | 11/33 (33.3) | 0.779 |
| Starting dose <420 mg, n (%) | 12 (50.0) | 25 (75.8) | 0.054 |
| Grade 3-5 adverse event, n (%) | 20 (83.3) | 21 (63.6) | 0.139 |
| Permanent discontinuation due to an AE, n (%) | 11 (45.8) | 6 (18.2) | 0.039 |
| Median potential administrative follow-up, days | 2,295.5 | 874 | - |

**B. Time-to-event sensitivity analyses**

| **Outcome** | **Events/N** | **HR, later vs earlier era (95% CI)** | **Cox p** | **Log-rank p** |
| --- | --- | --- | --- | --- |
| Permanent discontinuation | 17/57 | 0.54 (0.19-1.55) | 0.251 | 0.246 |
| First Grade 3-5 adverse event | 41/57 | 0.75 (0.41-1.39) | 0.364 | 0.363 |

Treatment-initiation dates ranged from March 2016 to February 2025 and were available for all 57 patients. Panel A presents n (%) unless otherwise stated; categorical variables were compared using Fisher's exact test. Potential administrative follow-up was calculated from initiation to December 1, 2025. Panel B presents univariable Cox proportional-hazards estimates with the earlier era as reference. Because subgroup sizes were small and the later era had shorter potential follow-up, all calendar-era analyses are exploratory. AE, adverse event; CI, confidence interval; CrCl, Cockcroft-Gault creatinine clearance; HR, hazard ratio.

**Supplementary Table S5. Patient-level paired analysis of temporal adverse-event patterns**

Patients were classified by whether they experienced at least one adverse event in each organ-system cluster during each treatment period.

**A. Equal-window sensitivity analysis: days 0-90 versus days 91-180 (N=47)**

Paired omnibus permutation test: statistic = 9.855; p = 0.2396; 100,000 permutations.

| **Adverse-event cluster** | **Days 0-90, n (%)** | **Days 91-180, n (%)** | **Exact McNemar p** | **Holm-adjusted p** |
| --- | --- | --- | --- | --- |
| Hematologic | 10 (21.3) | 6 (12.8) | 0.388 | 1.000 |
| Cardiac | 7 (14.9) | 2 (4.3) | 0.125 | 1.000 |
| Bleeding | 6 (12.8) | 3 (6.4) | 0.508 | 1.000 |
| Infectious | 7 (14.9) | 12 (25.5) | 0.302 | 1.000 |
| Hepatic | 0 (0.0) | 1 (2.1) | 1.000 | 1.000 |
| Gastrointestinal | 5 (10.6) | 2 (4.3) | 0.375 | 1.000 |
| Dermatologic | 4 (8.5) | 3 (6.4) | 1.000 | 1.000 |
| Metabolic | 0 (0.0) | 0 (0.0) | 1.000 | 1.000 |
| Renal | 1 (2.1) | 0 (0.0) | 1.000 | 1.000 |
| Neurologic | 0 (0.0) | 0 (0.0) | 1.000 | 1.000 |
| General | 2 (4.3) | 1 (2.1) | 1.000 | 1.000 |

**Supplementary Table S5 (continued)**

**B. Exploratory unequal-window analysis: days 0-90 versus the full period after day 90 (N=57)**

Paired omnibus permutation test: statistic = 24.288; p = 0.0046; 100,000 permutations.

| **Adverse-event cluster** | **Days 0-90, n (%)** | **After day 90, n (%)** | **Exact McNemar p** | **Holm-adjusted p** |
| --- | --- | --- | --- | --- |
| Hematologic | 13 (22.8) | 16 (28.1) | 0.678 | 1.000 |
| Cardiac | 9 (15.8) | 20 (35.1) | 0.035 | 0.312 |
| Bleeding | 11 (19.3) | 8 (14.0) | 0.607 | 1.000 |
| Infectious | 8 (14.0) | 19 (33.3) | 0.027 | 0.293 |
| Hepatic | 0 (0.0) | 6 (10.5) | 0.031 | 0.312 |
| Gastrointestinal | 6 (10.5) | 10 (17.5) | 0.424 | 1.000 |
| Dermatologic | 5 (8.8) | 11 (19.3) | 0.210 | 1.000 |
| Metabolic | 0 (0.0) | 2 (3.5) | 0.500 | 1.000 |
| Renal | 2 (3.5) | 0 (0.0) | 0.500 | 1.000 |
| Neurologic | 1 (1.8) | 1 (1.8) | 1.000 | 1.000 |
| General | 3 (5.3) | 7 (12.3) | 0.219 | 1.000 |

Values are the number and percentage of patients with at least one adverse event in the indicated cluster and period. Each patient contributed no more than once per cluster within each period. Cluster-specific comparisons used two-sided exact McNemar tests, with Holm adjustment across the 11 clusters within each panel. The omnibus test exchanged each patient's complete early/late cluster profile within patient. Panel A included patients observed and at risk through day 180. Panel B is exploratory because the post-90-day period was longer and exposure opportunities differed among patients. Individual adverse-event records were not treated as independent observations.
